# Providing HIV-related services in China for men who have sex with men 

**DOI:** 10.2471/BLT.15.156406

**Published:** 2016-01-22

**Authors:** Weibin Cheng, Yanshan Cai, Weiming Tang, Fei Zhong, Gang Meng, Jing Gu, Chun Hao, Zhigang Han, Jingyan Li, Aritra Das, Jinkou Zhao, Huifang Xu, Joseph D Tucker, Ming Wang

**Affiliations:** a Department of AIDS/STD Control and Prevention, Guangzhou Center for Disease Control and Prevention, No. 1 Qide Road, Jiahe, Guangzhou, Guangdong,510440, China.; b University of North Carolina Project-China, Guangzhou, China.; c Lingnan Partners Community Support Center (GZTZ.ORG), Guangzhou, China.; d School of Public Health, Sun Yat-sen University, Guangzhou, China.; e Fielding School of Public Health, University of California Los Angeles, Los Angeles, United States of America.; f Global Fund to fight AIDS, Tuberculosis and Malaria, Geneva, Switzerland.

## Abstract

**Problem:**

In China, human immunodeficiency virus (HIV) care provided by community-based organizations and the public sector are not well integrated.

**Approach:**

A community-based organization and experts from the Guangzhou Center for Disease Control and Prevention developed internet-based services for men who have sex with men, in Guangzhou, China. The internet services were linked to clinical services offering HIV testing and care.

**Local setting:**

The expanding HIV epidemic among men who have sex with men is a public health problem in China. HIV control and prevention measures are implemented primarily through the public system. Only a limited number of community organizations are involved in providing HIV services.

**Relevant changes:**

The programme integrated community and public sector HIV services including health education, online HIV risk assessment, on-site HIV counselling and testing, partner notification, psychosocial care and support, counting of CD4+ T-lymphocytes and treatment guidance.

**Lessons learnt:**

The internet can facilitate HIV prevention among a subset of men who have sex with men by enhancing awareness, service uptake, retention in care and adherence to treatment. Collaboration between the public sector and the community group promoted acceptance by the target population. Task sharing by community groups can increase access of this high-risk group to available HIV-related services.

## Résumé

**Problème:**

En Chine, la prise en charge du virus de l'immunodéficience humaine (VIH) par des organismes communautaires et par le secteur public n'est pas bien intégrée.

**Approche:**

Un organisme communautaire et des experts du Centre de contrôle et de prévention des maladies de Guangzhou ont mis au point des services sur internet pour les hommes ayant des rapports sexuels avec d'autres hommes, à Guangzhou, en Chine. Ces services internet étaient rattachés à des services cliniques proposant un dépistage et un traitement du VIH.

**Environnement local:**

La propagation de l'épidémie de VIH chez les hommes ayant des rapports sexuels avec d'autres hommes est un problème de santé publique en Chine. Les mesures de lutte et de prévention du VIH sont principalement mises en œuvre par le système public. Seul un petit nombre d'organismes communautaires offrent des services en lien avec le VIH.

**Changements significatifs:**

Le programme a intégré des services relatifs au VIH de la communauté et du secteur public, notamment des services d'éducation à la santé, l'évaluation en ligne des risques liés au VIH, le dépistage du VIH sur site et des conseils associés, l'information des partenaires, des soins et un soutien dans le domaine psychosocial, la numération des lymphocytes T CD4+ et des recommandations en matière de traitement.

**Leçons tirées:**

Internet peut faciliter la prévention du VIH chez une partie des hommes ayant des rapports sexuels avec d'autres hommes en renforçant la sensibilisation, l'utilisation de services, le maintien des soins et le respect du traitement. La collaboration entre le secteur public et le groupe communautaire a favorisé l'acceptation du projet par la population cible. Le partage des tâches entre groupes communautaires peut améliorer l'accès de ce groupe à haut risque aux services disponibles en matière de VIH.

## Resumen

**Situación:**

En China, el cuidado contra el virus de inmunodeficiencia humana (VIH) proporcionado por organizaciones de ámbito comunitario no está bien integrado con el sector público.

**Enfoque:**

Una organización de ámbito comunitario y expertos del Centro para el Control y la Prevención de Enfermedades de Guangzhou han desarrollado servicios en línea para hombres que mantienen relaciones sexuales con otros hombres en Guangzhou (China). Los servicios en línea estaban vinculados con servicios clínicos que ofrecían pruebas y tratamiento del VIH.

**Marco regional:**

La epidemia de VIH en expansión entre hombres que mantienen relaciones sexuales con otros hombres es un problema de salud pública en China. Las medidas de control y prevención del VIH se implementan principalmente a través del sistema público. Únicamente un pequeño número de organizaciones comunitarias proporcionan servicios para el VIH.

**Cambios importantes:**

El programa integró servicios comunitarios y del sector público para combatir el VIH, incluyendo educación sanitaria, evaluación de riesgos del VIH en línea, asesoramiento y pruebas del VIH in situ, notificación a la pareja, cuidado y apoyo psicosocial, recuento de linfocitos T CD4+ y guía de tratamiento.

**Lecciones aprendidas:**

Internet puede facilitar la prevención del VIH entre un subgrupo de hombres que mantienen relaciones sexuales con otros hombres mediante el aumento de la toma de conciencia, el uso de los servicios, la recepción de atención y el cumplimiento del tratamiento. La colaboración entre el sector público y el grupo comunitario fomentó la aceptación por parte de la población objetivo. El reparto de tareas entre los grupos comunitarios puede aumentar el acceso de este grupo en alto riesgo a los servicios relativos al VIH disponibles.

## ملخص

**المشكلة:**

لوحظ أن الرعاية المقدمة في الصين لمرضى فيروس العوز المناعي البشري من المنظمات المجتمعية والقطاع العام غير متكاملة على النحو الأمثل.

**الأسلوب:**

قام مسؤولون في إحدى المنظمات المجتمعية وخبراء من مركز "قوانغتشو" لمكافحة الأمراض والوقاية منها بإعداد الخدمات المرتكزة على شبكة الإنترنت للرجال الذين يقيمون علاقات جنسية مثلية "قوانغتشو" في الصين. وتم ربط خدمات شبكة الإنترنت بالخدمات السريرية التي توفر فحص فيروس العوز المناعي البشري ورعاية المصابين به.

**المواقع المحلية:**

إن وباء فيروس العوز المناعي البشري المنتشر بين الرجال الذين يقيمون علاقات جنسية مثلية هو مشكلة صحية عامة في الصين. يتم تنفيذ تدابير مكافحة فيروس العوز المناعي البشري والوقاية منه في الأساس من خلال النظام العام. لا يوجد سوى عدد محدود فقط من المنظمات المجتمعية المعنية بتقديم خدمات فيروس العوز المناعي البشري.

**التغيّرات ذات الصلة:**

قام البرنامج بدمج خدمات فيروس العوز المناعي البشري المقدمة من المجتمع والقطاع العام بما في ذلك التثقيف الصحي، وتقييم مخاطر فيروس العوز المناعي البشري على شبكة الإنترنت، وتقديم استشارات عن فيروس العوز المناعي البشري في الموقع وإجراء الفحص لاكتشافه، وإبلاغ الطرف الآخر، وتوفير الرعاية والدعم النفسي والمجتمعي، وإحصاء عدد خلايا اللمفاويات التائية CD4+، وتقديم إرشادات للعلاج.

**الدروس المستفادة:**

يمكن لشبكة الإنترنت المساهمة في تسهيل الوقاية من فيروس العوز المناعي البشري بين مجموعة فرعية من الرجال الذين يقومون بعلاقات جنسية مثلية من خلال زيادة الوعي، واستيعاب الخدمات، واستبقاء الرعاية، والالتزام بالعلاج. وأدى التعاون بين القطاع العام والمجموعة المجتمعية إلى تشجيع قبول المرضى من جانب قطاع السكان المستهدفين. إن مشاركة المهام من جانب الجماعات المجتمعية يمكن أن يزيد من نسبة حصول هذه المجموعة المعرضة للخطر على الخدمات المتوفرة المرتبطة بفيروس العوز المناعي البشري.

## 摘要

**问题:**

在中国，社区组织和政府机构提供的人体免疫缺陷病毒 (HIV) 预防和关怀服务未充分整合。

**方法:**

广州的社区组织和广州市疾病预防控制中心的专家为男男性行为者创建了基于互联网的 HIV 服务。互联网的服务与现场 HIV 检测和关怀服务形成无缝连接。

**当地状况:**

男男性行为者群体 HIV 的疫情逐渐上升是中国当前面临的一大公共卫生问题。HIV 防控措施主要通过政府系统实施，仅有少数社区组织参与提供 HIV 服务。

**相关变化:**

该计划将社区组织与政府机构的 HIV 服务结合起来。这些服务包括健康教育、在线 HIV 风险评估、HIV 现场咨询与检测、性伴通知、社会心理关怀和支持、CD4+ T 淋巴细胞计数监测和抗病毒治疗指导。

**经验教训:**

通过互联网提高人群风险意识、服务渗透率、随访维持率和治疗依从性，能够在部分男男性行为者中促进 HIV 预防。政府机构和社区组织之间的合作提升了目标人群的接受度。社区组织的参与让高风险群体能够更加方便地接受现有的 HIV 相关服务。

## Резюме

**Проблема:**

В Китае существует проблема недостаточной интеграции медицинских услуг, оказываемых местными общественными организациями и государственным сектором, для лиц, зараженных вирусом иммунодефицита человека (ВИЧ).

**Подход:**

Организацией на уровне общин и специалистами Центра по борьбе с болезнями и их профилактике г. Гуанчжоу были разработаны интернет-службы для мужчин, практикующих секс с мужчинами, в г. Гуанчжоу, Китай. Интернет-службы были привязаны к медицинским услугам, предполагающим проведение тестирования на ВИЧ и предоставление связанного с этим вирусом лечения.

**Местные условия:**

Растущая эпидемия ВИЧ среди мужчин, практикующих секс с мужчинами, представляет собой проблему общественного здравоохранения в Китае. Меры по борьбе с ВИЧ и его профилактике реализуются в основном через государственную систему. Местные общественные организации вовлечены в оказание услуг по ВИЧ лишь в ограниченном количестве.

**Осуществленные перемены:**

С помощью программы была осуществлена интеграция услуг, связанных с ВИЧ, предоставляемых местными общественными организациями и государством, включая медико-санитарное просвещение, оценку риска заражения ВИЧ в онлайн-режиме, консультирование и тестирование на ВИЧ на месте, уведомление партнера, психосоциологическую помощь и поддержку, определение количества T-лимфоцитов с фенотипом CD4+ и консультации по лечению.

**Выводы:**

С помощью Интернета можно упростить профилактику ВИЧ среди подгруппы мужчин, практикующих секс с мужчинами, за счет повышенной информированности, увеличенного количества оказываемых услуг и более строгого соблюдения больными предписаний врачей, в том числе больничного режима. Благодаря взаимодействию государственного сектора и общин вырос показатель использования услуг целевой группой населения. Распределение обязанностей между местными общественными группами может поспособствовать увеличению доступности имеющихся услуг, связанных с ВИЧ, для данной группы повышенного риска.

## Introduction

Community engagement is important for controlling the human immunodeficiency virus (HIV) epidemic among men who have sex with men (MSM).^1^^–^^3^ In China, not-for-profit community-based organizations (CBOs) are engaged with the social, educational, environmental or public safety needs of the community. However, the majority of public sector-funded HIV programmes in China have failed to engage CBOs^4^^,^^5^ and have had limited success in preventing HIV.^6^ Furthermore, the lack of endorsement for CBOs from the public sector has hampered the work of CBOs on HIV control and delivery of related services. Most MSM-friendly CBOs do not offer services like HIV testing, post-test counselling, result notification and follow-up, which limit their ability to provide comprehensive care services.

To address these problems, an HIV care and prevention programme sponsored by the Bill & Melinda Gates Foundation was launched in China in 2008. The programme promoted collaboration between public sector agencies and CBOs in the delivery of prevention and support services. Preventive services were directed to high-risk groups and included reducing risk behaviours and increasing HIV testing. Support services focused on increasing access to care and improving the quality of services for people living with HIV. During the programme, the responsibility for some HIV-targeted interventions began to shift from the public sector to CBOs.^7^

Here we describe a project collaboration called IMPACT (integration minimum package of prevention in accelerating case finding and treatment) in Guangzhou.

## Local setting

In China, basic HIV control and prevention measures are implemented primarily through the public health system organized by the Chinese Center for Disease Control and Prevention (CCDC). These measures include HIV-testing campaigns, condom promotion, behavioural change interventions, follow-up care for people living with HIV and implementation of free antiretroviral therapy (ART).^4^^,^^5^ Due to stigma and discrimination against homosexuality and people living with HIV, MSM are usually hard to reach. Most of the CBOs working with MSM are newly-established organizations that know the community very well. However, a large proportion of CBOs have not been well supported by the public health sector due to policy barriers and a perceived lack of expertise in HIV prevention in these organizations. Many CBOs have not survived due to lack of funds.

In 2011, MSM constituted almost one-third of the 48 000 new HIV infections.^5^ The HIV prevalence among MSM has increased from 2.5% in 2006 to 7.4% in 2009.^8^^,^^9^ Furthermore, it is estimated that in 2011, 50% of MSM who were HIV-positive did not know their status.^5^

Guangzhou is a city in southern China with over 12 million inhabitants. In 2008, it was estimated that 44 593 sexually active MSM were living in the city. HIV prevalence among MSM has increased significantly from 5.0% (19/379) in 2008 to 11.4% (72/633) in 2013.^10^

## Relevant changes

Previously CBOs were restricted to carrying out programme implementation only. Here, the Guangzhou CDC and the Lingnan Partners Community Support Center worked together to design an integrated service including HIV health education, online HIV-risk assessment, on-site HIV counselling and testing, partner notification, psychosocial care and support, CD4+ T-lymphocyte count testing and guidance on clinical treatment. Each component of the project was designed with a specific goal and relevant HIV care service (Table 1). We describe these components below.

**Table 1:** HIV-related services for men who have sex with men provided by collaboration between the public sector and a community-based organization in Guangzhou, China, 2008–2013

Type of service	Goal	Content	Service package (year available)	Related information
Internet-based prevention services	To assist MSM in generating, reinforcing, and validating awareness of HIV risk and safe sex behaviour	HIV health education and HIV testing mobilization	Online HIV knowledge dissemination (2008)	http://www.gztz.org
			Scenario-based application (2010)	http://www.gztz.org Only open for registered users
			Online HIV risk self-assessment system (2012)	http://pink.gztz.org Only for registered users
			Health-related online broadcasting for the gay community (2013)	WeChat, a free social media application. WeChat ID: Lingnan-station
Online-to-off line service linkage	To increase connectivity between online and offline services and promote HIV testing among partners of newly diagnosed HIV individuals	HIV testing and counselling booking and result notification and counselling	Online HIV testing appointment system (2010)	http://lingnan.gztz.org
			Online testing-results-notification system (2010)	http://www.gztellthem.org
			Anonymous partner notification system (Easy Tell®, 2009)	http://www.gztellthem.org
Offline one-stop shop service	To boost the confidence among MSM in receiving the service and to keep the person in care	On-site HIV counselling and testing, psychosocial care and support for HIV, ART support	HIV rapid testing (2011)	http://lingnan.gztz.org/May/UserStory
			CD4+ T-lymphocyte count and viral load testing, ART support (2010)	NA
			One-on-one care support for newly diagnosed individuals (2009)	NA

ART: antiretroviral therapy; CD4: cluster of differentiation 4; HIV: human immunodeficiency virus; MSM: men who have sex with men; NA: not applicable.

### Project components

#### Online prevention tools

We developed two internet tools: a scenario-based application and an HIV risk self-assessment system. The scenario-based application is an interactive internet application that simulates real-life HIV risk scenarios. The objective of this application is to encourage HIV testing and reduce high-risk behaviours.

The online HIV risk self-assessment system calculates an individualized HIV-risk score by evaluating an individual’s risk profile. Based on the results, this system also provides tailored guidance to promote HIV testing and behavioural change. These two online tools reach a wide user base via the internet and provide tailored interventions to meet specific needs. HIV education was also provided online and by public service broadcasting via a social media application (WeChat).

#### Online-to-offline service

This component linked virtual interventions for increasing HIV testing to actual HIV testing and facilitated HIV care. The online prevention tools described above were linked to an online appointment system for HIV testing. People could choose to have a test at one of three facilities in the area and test results were made available via an online notification system. A person who made an online appointment could also choose that the notification system (Easy Tell®) informed their partner anonymously about a positive result by clicking a consent button and providing the partner’s mobile phone number or email address.^11^ If the result was positive, a system message which contained a verification code was sent automatically to the partner. Notified partners could retrieve the information through the platform using the verification code and then be linked to HIV testing from the platform.

#### Service centre

We set up a one-stop service centre in Guangzhou, which was coordinated by a local CBO and Guangzhou CDC. In this centre, public sector staff provided on-site blood sampling and testing and carried out epidemiological investigations, such as HIV sentinel surveillance among MSM and medical follow-up for people who tested positive. People who were tested were also asked questions about their sexual behaviour. Meanwhile, CBO peers delivered high-quality and timely pre- and post-test counselling, psychosocial support services, guidance on retention in care and ART adherence support services. The quality of these processes was ensured by following a stringent selection process. Peer workers were trained by staff from Guangzhou CDC and the CBO for about three months in the one-stop service centre. All peer counsellors signed a confidentiality agreement.

### Project outcome

**Fig. 1:**
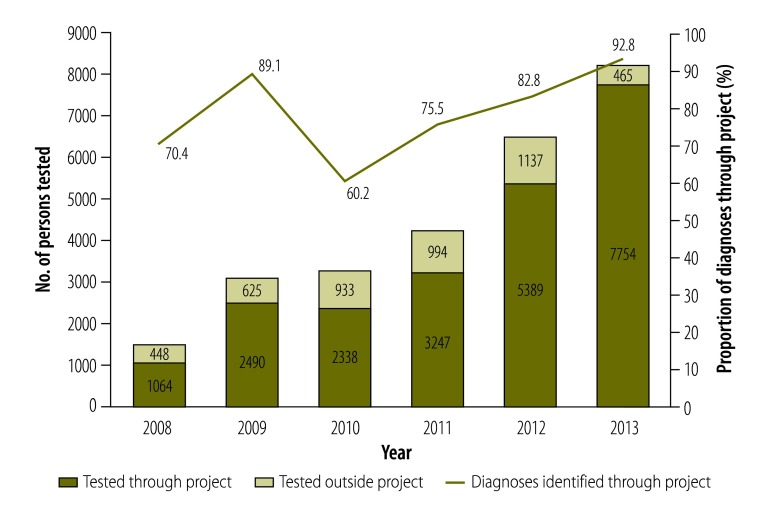
HIV testing of men who have sex with men through a project linking internet-based HIV services to testing, in Guangzhou, China, 2008–2013

Between 2008 and 2013, the project gave 22 282 HIV antibody tests, of which 999 tests were positive. The annual number of tests increased from 1064 in 2008 to 7754 in 2013. By 2013, tests conducted under the project accounted for more than 80% of total HIV tests (22 282/26 884) and new HIV diagnoses (999/1218) among MSM in Guangzhou (Fig. 1). Currently, an average of 25 people make appointments and get tested through the project each day. This project has addressed the needs of this community and has been improving access to HIV services.

HIV: human immunodeficiency virus.

The project also ensured continuum-of-care services, including linkage to care, retention in care, ART initiation and ART adherence. Of the 999 HIV-positive people, it was possible to link 948 (95%) to care services, while 891 (94%) of those linked were successfully retained in care. Among those who were retained in care and met the criteria for receiving free ART (CD4+ count < 350 cells/μL), over 85% (353/415) initiated ART. Based on the percentage of people on ART, we assumed that approximately 75% of these people achieved viral suppression (less than 50 copies/mL).

## Challenges and lessons learnt

The project had several limitations and faced several challenges (Box 1). One of the key limitations is that this project did not collect comprehensive pre-intervention data to demonstrate the effect of the intervention. Also, the project could recruit only a subset of MSM residing in the study area and found it difficult to reach out to some subgroups (e.g. older MSM and rural MSM). These hard-to-reach subgroups reportedly have lower levels of education, poorer HIV knowledge and fewer opportunities to access HIV-related services, making them highly vulnerable to HIV.^10^

Box 1 **Summary of main lessons learnt**

- The internet can facilitate human immunodeficiency virus (HIV) prevention among a subset of men who have sex with men by enhancing awareness, service uptake, retention in care and adherence to treatment.
- Collaboration between the public sector and the community group promoted acceptance by the target population.
- Task sharing by community groups can increase access of this high-risk group to available HIV-related services.

Despite the expanding scope of HIV services arising from collaboration between the public sector and the CBO, the relative absence of formal partnerships with treatment facilities limited the extent to which this intervention impacted clinical management and retention. Closer partnerships with clinical facilities may further enhance the project. Our annual HIV sentinel surveillance face-to-face survey showed that 80% (2081/2603) of respondents received some form of HIV-related service during the past year, HIV testing coverage remained relatively low (47%; 1227/2603) among MSM.^12^^,^^13^ This disparity indicates the need to strengthen the promotion of HIV testing. Other challenges included instability of peer workforce, lack of sustained funding and intervention information fatigue.

Despite the limitations, the high percentage of people who were retained in HIV care suggests that collaboration between the public sector and CBOs can be successful in providing high-quality HIV-related services. Internationally, task-shifting from health professionals to CBOs has proven to be effective in the provision of counselling, testing, care and treatment services for HIV.^7^^,^^14^ In the current project, the CBO engaged with the MSM community and the public sector agencies contributed technical proficiency and worked together to improve the quality of services offered. The key to this successful collaboration was the mutual trust between the public sector and the CBO. The role of the CBO was not restricted to programme implementation; it was also involved in the project development phases.^15^ The one-stop shop concept of providing a range of HIV services from a single location increased retention in HIV care.

The project may be usefully adapted to other places in China and perhaps other low- and middle-income countries, where opportunities for community engagement are limited. Finally, experience gained from the project can also inform decision-making in other public health domains, which is likely to benefit from increased collaboration between the public sector and community groups.
